# Impact of novel agent therapies on immune cell subsets and infectious complications in patients with relapsed/refractory multiple myeloma

**DOI:** 10.3389/fonc.2023.1078725

**Published:** 2023-04-21

**Authors:** Lukas John, Kaya Miah, Axel Benner, Elias K. Mai, Katharina Kriegsmann, Michael Hundemer, Dorothee Kaudewitz, Carsten Müller-Tidow, Karin Jordan, Hartmut Goldschmidt, Marc S. Raab, Nicola Giesen

**Affiliations:** ^1^ Department of Medicine V - Hematology, Oncology and Rheumatology, University Hospital Heidelberg, Heidelberg, Germany; ^2^ Clinical Cooperation Unit Molecular Hematology/Oncology, Department of Internal Medicine V, Heidelberg University Hospital, and German Cancer Research Center (DKFZ), Heidelberg, Germany; ^3^ Division of Biostatistics, German Cancer Research Center (DKFZ), Heidelberg, Germany; ^4^ National Center for Tumor Diseases (NCT), Heidelberg, Germany; ^5^ Department of Hematology, Oncology and Palliative Medicine, Ernst von Bergmann Hospital, Potsdam, Germany

**Keywords:** CD4+-T-cells, infections, relapsed refractory multiple myeloma, novel agents, immune cell subsets

## Abstract

**Introduction:**

Infections are a leading cause of morbidity and mortality in patients with multiple myeloma (MM).

**Methods:**

To examine the effects of modern second-generation novel agent therapy on immune cell subsets, in particular CD4+-T-cells, and infectious complications in patients with relapsed/refractory MM (RRMM), we conducted a prospective cohort study in 112 RRMM patients.

**Results:**

Substantially decreased CD4+-T-cells <200/µl before initiation of relapse therapy were detected in 27.7% of patients and were associated with a higher number of previous lines of therapy. Relapse therapy with carfilzomib or pomalidomide showed a significant further decrease of CD4+-T-cells. All novel agents led to a significant decrease of B-cell counts. Overall, infections were frequent with 21.3% of patients requiring antibacterial therapy within the first 3 months of relapse therapy, 5.6% requiring hospitalization. However, in the setting of standard antimicrobial prophylaxis in RRMM patients with very low CD4+-T-cells, no significant association of CD4+T-cell count and an increased risk of infection could be detected.

**Discussion:**

Our findings imply that reduced CD4+-T-cell numbers and infections are common in patients with RRMM. We also demonstrate an association with the number of previous therapies and certain substances suggesting an increased need for personalized prophylaxis strategies for opportunistic infections in this patient cohort.

## Introduction

Infections are a leading cause of morbidity and mortality in multiple myeloma (MM) (1–5). MM itself is associated with a significant risk for infectious complications (3) due to changes in the immune system, such as hypogammaglobulinemia and defects in the cellular immune response (5, 6). MM patients therefore have a sevenfold higher risk of infection compared to the standard population (2, 7) and suffer more complications from infections (8). Particularly in later stages of the disease, they have an increased risk for opportunistic infections such as invasive fungal infections, cytomegalovirus (CMV)- and herpes zoster reactivations as well as pneumocystis pneumonia (PcP) (6, 9–11). They also have a higher risk for complications associated with COVID-19 (12–14), as well as a decreased response to vaccination against COVID-19 and other diseases (15–20). Although novel agent-based therapies have dramatically increased the life expectancy of patients with MM (21), they further exacerbate immunosuppression (2, 7, 22, 23). Despite the high prevalence of infections in MM patients, only few predictive risk factors have so far been identified (24). In particular little is known about the effects of prolonged and continuous treatment with novel agents in patients with relapsed or refractory MM (RRMM). While severe and prolonged neutropenia (<500/µl) is an established risk factor for fever of unknown origin in cancer patients, MM patients receiving novel agents often suffer from infectious complications outside the setting of severe neutropenia (5). Novel predictors of infections in this setting are therefore urgently needed.

CD4+-T-cells are a central part of the adaptive immune system. While the number of CD4+-T-cells in the peripheral blood has originally been linked to a high risk for the development of acquired immunodeficiency syndrome (AIDS) in HIV-infected patients and is used as a marker for stages in AIDS (24, 25), reduced CD4+-T-cells have also been shown to be a marker for the risk of PcP in non-HIV-infected patients (26, 27). Furthermore, lower CD4+-T-cells are known to be a risk factor for the development of herpes zoster virus reactivation in MM patients receiving bortezomib, as well as for opportunistic infections in patients with MM receiving conventional therapy (6, 28). This led to some guidelines recommending PcP- and herpes zoster prophylaxis in MM patients showing CD4+-T-cell-numbers <200/µl (29). Interestingly, it has been shown in the COVID-19 pandemic that decreased CD4+-T-cells are a risk factor for a more severe course of the disease (30, 31), therefore suggesting they might also facilitate non-opportunistic infections. The depletion of other immune cells such as NK-cells (32) and CD8+-T-cells (33, 34) was also linked to an increased risk of infections. Despite their importance, however, there is little data on the effects of novel agent-therapy on CD4+-T-cell and other immune cell numbers for RRMM patients outside the setting of clinical trials. This holds particularly true for second generation novel substances such as daratumumab, carfilzomib and pomalidomide.

The aim of this prospective cohort study was to prospectively examine the distribution of immune cell subsets, in particular CD4+-T-cell numbers, in a real-life cohort of patients with RRMM receiving treatment with second generation novel agents at our institution and the effects of novel agent treatment on these subsets. As a second exploratory objective we correlated CD4+-T-cell numbers with the frequency of infections to assess a possible impact on the risk of infections.

## Materials and methods

### Study population

**Figure 1:**
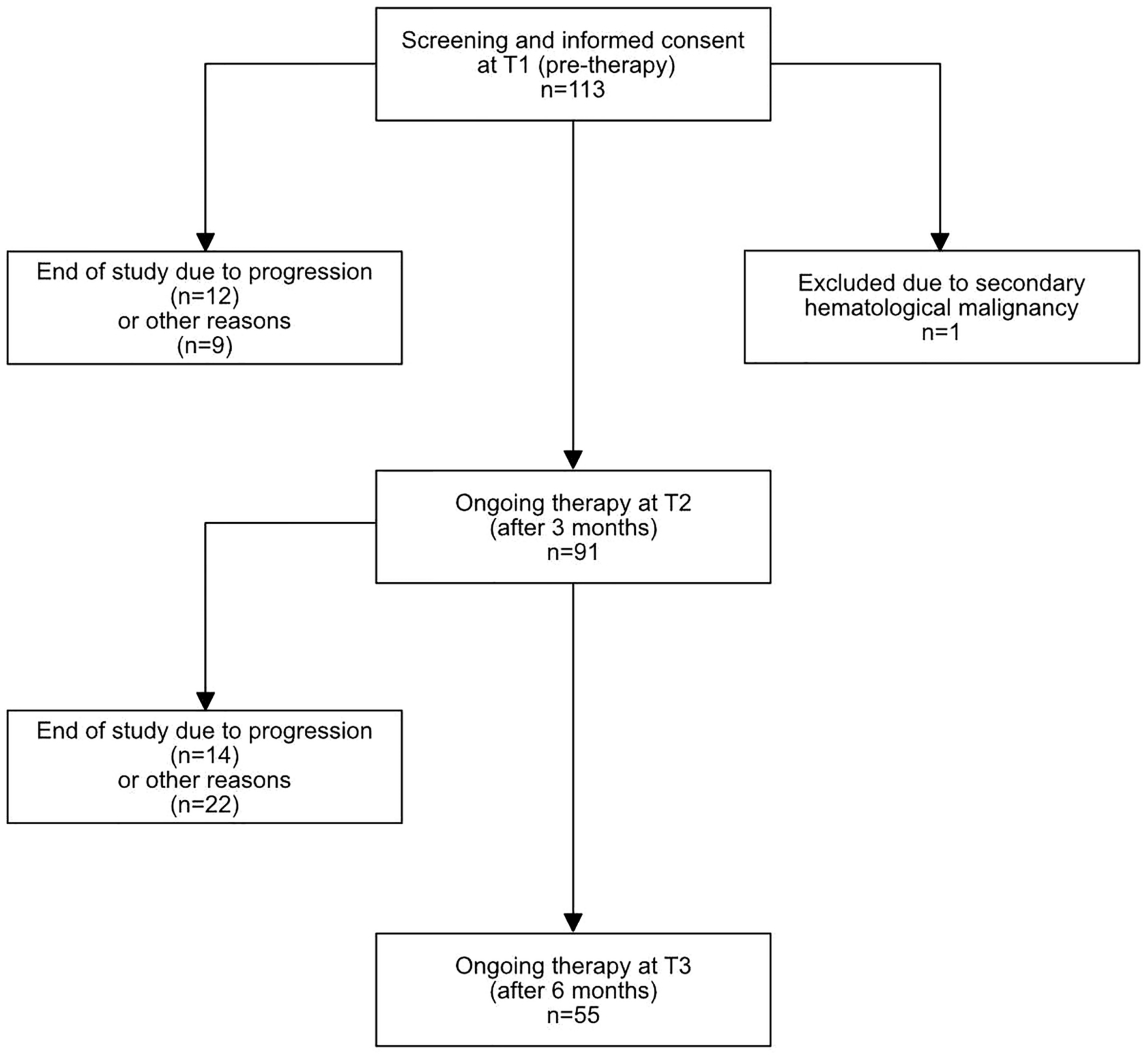
Flow diagram of included patients. 113 patients were initially included, 1 patient was excluded due to a secondary hematological malignancy. At T2, 91 patients had ongoing therapy, and 55 at T3. The exact reasons for study discontinuation are described in **Table S2**.

We prospectively included 113 patients with RRMM at the initiation of relapse treatment with a systemic combination therapy containing second-generation novel agents (carfilzomib, pomalidomide, elotuzumab, daratumumab) at the University Hospital Heidelberg, a tertiary university referral center, between November 2018 and September 2020. Exact treatment combinations are shown in **Table S1**. Patients with secondary hematological malignancies or with secondary solid malignancies requiring chemotherapy were not included in this study. One patient was excluded from the final study population (total n=112), due to the presence of B-chronic-lymphocytic leukemia as an additional hematological malignancy affecting peripheral lymphocyte cell counts and immune function (**Figure 1**).

### Data collection

Data was collected before the start of relapse therapy (T1), after 3 months (T2) and after 6 months of therapy (T3). At each timepoint, a detailed medical history of infectious complications within the last 3 months including the number, type and duration of infections as well as accompanying symptoms and treatment received for infections was conducted for all patients. All patients also received a differential blood count with fluorescence-activated cell sorting (FACS)-based subtyping of lymphocytes (CD4+-T-cells, CD8+-T-cells, NK-cells and B-cells). Immunoglobulin-levels (IgG, IgA, IgM) as well as myeloma activity (monoclonal protein, free light chains in serum and light chains in urine) were determined at baseline and all subsequent follow-ups. Corrected IgG was determined by multiplying the non-monoclonal part of the gamma-fraction in serum electrophoresis and total plasma protein. In patients with IgA myeloma and a monoclonal gradient in the gamma-fraction, uncorrected plasma IgG levels were used. Hypogammaglobulinemia was classified into three categories: mild, moderate and severe, as previously described (35). Infections were graded according to the common terminology criteria for adverse events (CTCAE) v.5.0.

### Standard of care anti-infective prophylaxis strategies

Patients who had CD4+-T-cell-numbers below 200/µl were prescribed PcP-prophylaxis with sulfomethoxazole/trimethoprime (cotrimoxazole) 960mg three times a week and anti-zoster prophylaxis with acyclovir 400mg bid according to national guidelines (29, 36). In addition, acyclovir was routinely prescribed in patients receiving proteasome inhibitors (PI) and/or daratumumab (20). Routine antibacterial prophylaxis using ciprofloxacin (500mg bid) or cotrimoxazole (960mg bid) was recommended for the first cycle of therapy, regardless of the type of administered therapy.

### FACS analysis

Lymphocyte subsets, i.e. percentages and absolute counts of T-, B-, and natural killer- (NK) cells as well as the CD4+- and CD8+- subpopulations of T-cells in peripheral blood, were measured with a 6-color TBNK Reagent with BD Trucount™ Tubes kit (BD, Heidelberg, Germany; containing CD3-FITC, CD16-PE and CD56-PE, CD45-PerCP-Cy5.5, CD4 PE-Cy7, CD19 APC, CD8 APC-Cy7). Measurements were performed on a BD FACSLyric™ flow cytometer.

### Data analysis

Frequency distributions of baseline patient characteristics, prior therapies, response assessments, CD4+-T-cell counts, and infections categorized by CTC grade were summarized descriptively.

In order to examine influences on immune cell subpopulations before treatment, multivariable analyses for linear CD4+-T-cell values at T1, T2 and T3 were carried out by utilizing regression models adjusting for the covariates age, number of prior therapies and therapy within the last 6 months. Logarithmized cell numbers were used to account for the high skewness in CD4+-T-cells. Estimated effects are reported along with 95% confidence intervals (CI95).

The effect of novel agents on immune cell subsets was assessed using Wilcoxon’s signed rank test for comparisons between T1 and T2 and the Friedman test for comparisons between the three timepoints T1, T2 and T3. In addition, a multivariable linear regression model including initial CD4+-T-cells (log10-transformed), daratumumab, pomalidomide and carfilzomib as covariates was fitted to assess the effect of individual substances on log10-transformed CD4+-T-cell numbers at T2.

To assess the effect of clinical covariates in infections, we fitted a multivariable continuation ratio model adjusting for the covariates of absolute CD4+-T-cell counts, age, number of prior therapies and dichotomized corrected IgG values. Estimated effects are reported along with corresponding 95%-confidence intervals. The single covariate effects were inferentially assessed by Wald tests.

All analyses are exploratory. Thus, no adjustment for multiple testing was done. Reported p-values are two-sided and considered to be statistically significant if p ≤0.05. The statistical analyses were performed using R version 4.1.2 (www.r-project.org) (37).

### Ethical approval

Written informed consent was obtained from all study participants. This study was approved by our institutional ethics review board (no. S-096/2017).

## Results

### Patient characteristics

A total of 112 patients were included in the analysis population before initiation of relapse therapy with second-generation novel agents. The median age was 69 years (range 42-90). 56.2% of patients were male, 43.8% female. The median number of previous therapies was 2 (range 1-13). 37.2% of patients had received 3 or more previous lines of therapy. An overview on patient characteristics is shown in **Table 1**.

**Table 1:** Patient characteristics.

Variable, n (%) If not other identified	Total
Total number of patients	112 (100%)
Age (median, range)	69 (42-90)
Sex: female	49 (43.8%)
Time from initial diagnosis in months (median,range)	61 (7-290)
Myeloma isotype	
IgG	67 (59.8%)
IgA	22 (19.6%)
free light chain	14 (12.5%)
other	9 (8.0%)
Prior lines of therapy, median (range)	2 (1-13)
Systemic therapy within the last 6 months	71 (63.4%)
Autologous transplant in history	87 (77.7%)
PI exposed	110 (98.2%)
Imid exposed	98 (87.5%)
CD38 exposed	34 (30.4%)
Double refractory	30 (26.8%)
Triple refractory	19 (16.7%)

Overview on characteristics of patients included in the study.

63.4% of patients had received systemic anti-myeloma therapy within the last 6 months, while the rest had been off systemic treatment for at least 6 months before the re-initiation of treatment. Follow-up information at T2 was available for 91 patients, and at T3 for 55 patients. The most common reason for loss of follow-up between T1 and T2 was progression or death due to progression (12/21, 57%). Between T2 and T3, 36 patients were lost to follow-up. From these, 14 patients (38.9%) changed therapy or died due to progression while one patient died due to an infection. An exact overview on reasons for discontinuation and loss of follow up can be found in **Table S2**.

### Treatment overview

All patients in the study received relapse therapy with at least one of the second-generation novel agents pomalidomide, carfilzomib, elotuzumab and/or daratumumab. Daratumumab was most frequently used, with 57.1% of patients receiving it. After 3 months (T2), 91 (81.4%) patients still received their initial therapy. Of those patients, 55 (60.4%) patients continued to receive combinations containing daratumumab, 27 (29.7%) received combinations containing carfilzomib and 19 (20.9%) and 8 (9.9%) received combinations containing pomalidomide or elotuzumab, respectively. Out of these patients, 9 (9.9%) received doublet combinations while 81 (89.0%) received triplet combinations. 1 patient (1.1%) received a quadruplet combination. The exact treatment combinations at each timepoint are shown in **Table S1**.

With regard to antibiotic and antiviral prophylaxis, out of 91 patients with follow-up at T2, 78 (84.5%) received anti-zoster prophylaxis. 42 patients (45.7%) received PcP-prophylaxis. 24 patients (26.1%) received antibiotic prophylaxis over the full three months of therapy. Between 3 and 6 months, proportions were very similar, except for continued antibiotic prophylaxis being less common, with only 7.4% of patients receiving it. At T2, 10 patients (11%) received intravenous immunoglobulin substitution and 4 patients (7.3%) received it at T3. An overview is shown in **Table S3**.

### Infectious complications

91 patients were evaluable for infections between T1 and T2. Within the first three months of relapse therapy (T1-T2), there were 19 infections with CTC grade ≥2, 3 infections with CTC Grade 3 and 2 infections with CTC Grade 4.

Between months 4-6 of relapse therapy (T2-T3), 56 patients were evaluable for infections. There were a total of 9 infections ≥ CTC grade 2 during this period with 5 infections CTC grade 2, 1 infection CTC grade 3, 2 infections CTC grade 4 and one death due to pneumonia caused by influenza.

Of note, only one opportunistic infection (herpes zoster-reactivation) occurred during the observation period. This happened in a patient who did not receive anti-zoster-prophylaxis.

This confirms that serious infections are common in RRMM patients with 19 patients (21.3%) requiring antibiotics within the first three months of therapy and 5 (5.6%) requiring hospitalization.

### Analysis of CD4+-T-cells at initiation of relapse therapy

The median CD4+-T-cell count before the start of relapse therapy was 316.5/µl. Notably, even before the start of relapse therapy, 86 patients (76.8%) had reduced CD4+-T-cell-numbers <500/µl and 31 (27.7%) even had CD4+-T-cell-numbers <200/µl, the latter thus requiring specific anti-infective prophylaxis according to national guidelines (29). Notably, in 105 out of 112 patients, for which absolute neutrophil counts were available, no patient showed severe neutropenia <0.5/nl (median 3.2/nl, range: 0.77/nl-10.27/nl). 102 (97.1%) patients had neutrophil counts > 1/nl.

In order to identify risk factors for suppressed CD4+-T-cell numbers at onset of relapse therapy, we performed multivariable regression analysis considering the effects of age, the number of previous therapies, the administration of a preceding systemic therapy within the last 6 months (yes/no) and the type of this therapy (antibody, Imid, PI or others) on log-scaled CD4+-T-cells. We observed that a directly preceding therapy (coefficient estimate -0.17; CI95 [-0.29;-0.06]; Wald p=0.01) as well as the number of previous lines of therapy were significantly associated with a decrease in CD4+-T-cell numbers (coefficient estimate -0.03; CI95 [-0.06;-0.01]; Wald p=0.02). Age as another factor remained non-significant. In conclusion, we demonstrate that a majority of RRMM patients show severely depleted CD4+-T-cells, with both preceding therapy within the last 6 months and the amount of previous therapy lines being significant contributors (**Tables 2**, **S4**).

**Table 2:** Results of the fitted regression model for log10 CD4+-T-cell counts at T1.

	Units	Coefficient	CI95	p-value
(Intercept)		2.58	[2.14;3.01]	< 1e-04
Age		0.00	[-0.01;0.01]	0.71
Active therapy within the last 6 months	no yes	Ref -0.17	[-0.29;-0.06]	0.01
Number of previous therapies		-0.03	[-0.06;-0.01]	0.02

Active therapy within the last 6 months is associated with decreased CD4+-T-cell numbers(coefficient = -0.17; p=0.01), as are the number of previous lines of therapy (coefficient = -0.03; p=0.02). VIF are shown in **Table S4.**

### Development of CD4+-T-cells during relapse therapy

**Figure 2:**
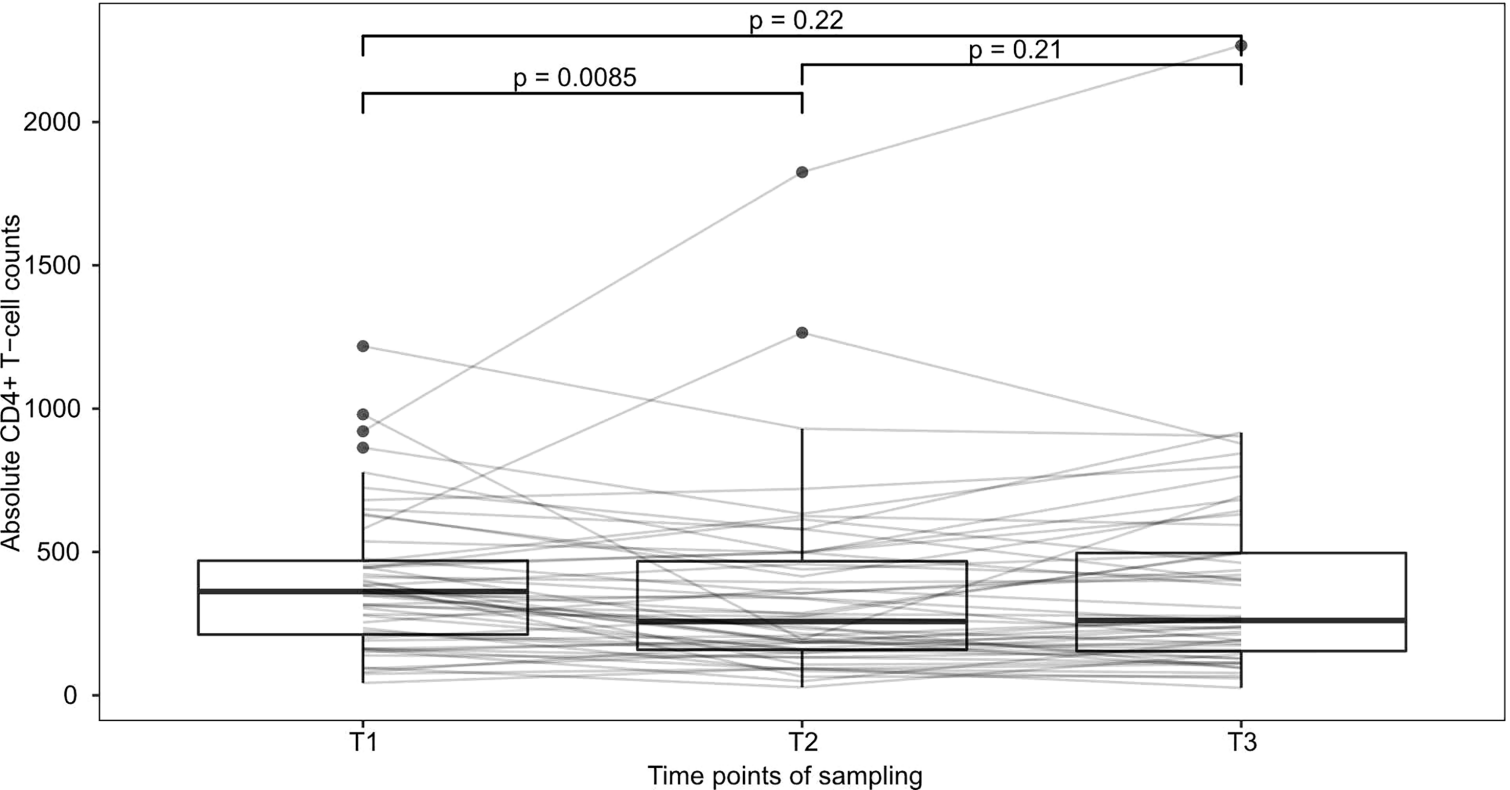
Development of CD4+-T-cells during therapy This figure shows patients with observed values at all 3 timepoints (n=52). Median CD4+-T-cell numbers declined from 362/µl at T1 to 257.5/µl at T2 and 261/µl at T3. p-values were calculated using Wilcoxon’s signed rank test and are shown above brackets.

To examine the effect of relapse therapy in general on CD4+-T-cell numbers, we first compared patients with measurements at all 3 timepoints (n=52). Median CD4+-T-cell numbers were 362/µl at T1, 257.5/µl at T2 and 261/µl at T3. CD4+ T-cell counts significantly declined under therapy between T1 and T2 (Wilcoxon signed p=0.009), however no further deterioration at T3 compared to T2 was seen (Wilcoxon signed p=0.21) implying that the strongest effect of therapy is seen between T1 and T2 (**Figure 2**).

**Figure 3:**
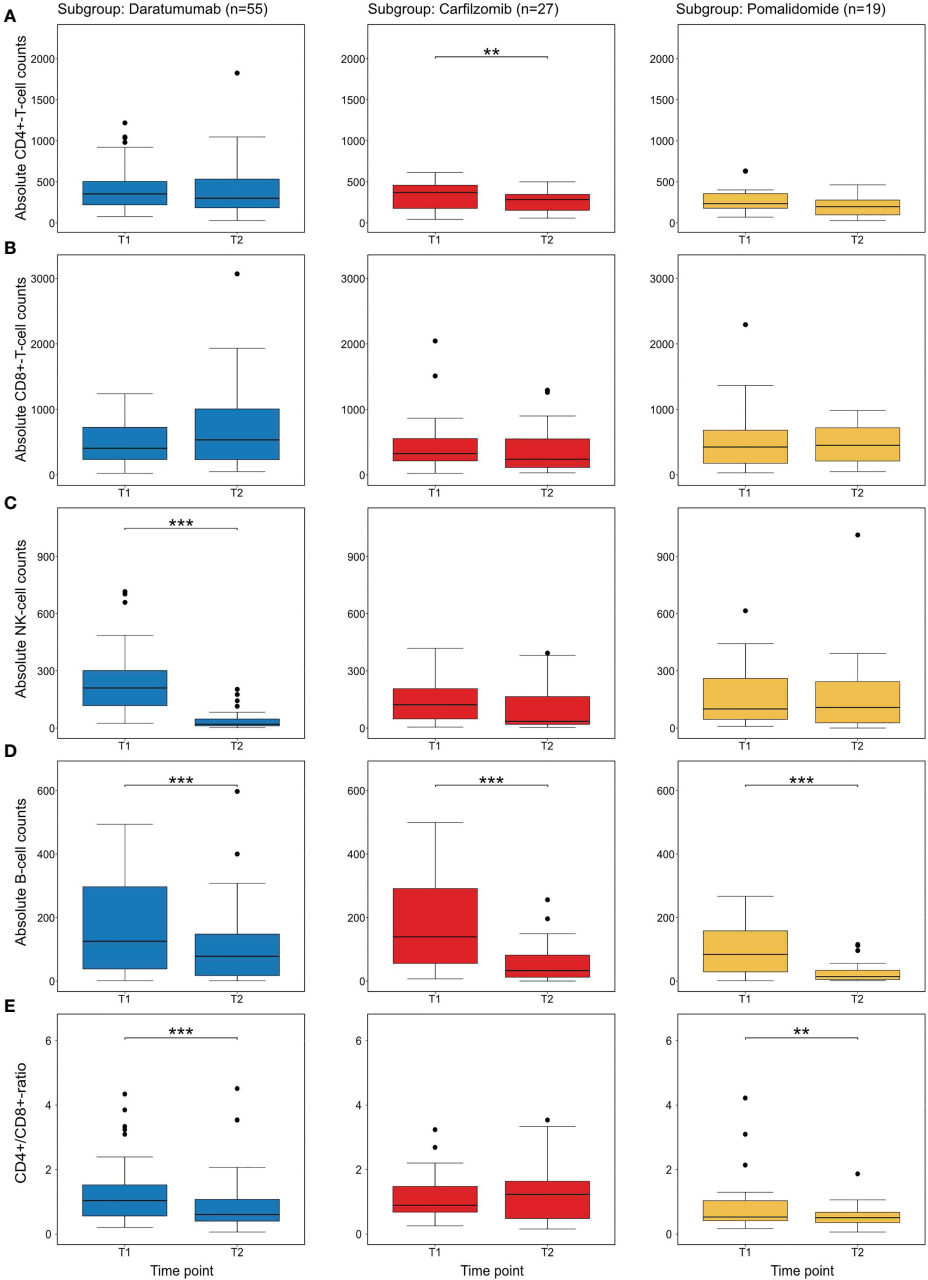
Descriptive analysis of individual novel agents on immune cell subsets at T2 **(A)** shows the distribution of CD4+-T-cell numbers before the initiation of therapy (T1) and after 3 months (T2) in treatment subgroups. In this univariate analysis only carfilzomib shows a statistically difference between CD4+-T-cell numbers. **(B)** shows CD8+-T-cell numbers with no substance showing a significant difference. **(C)** shows NK-cell numbers. As previously described (32), daratumumab significantly reduced NK-cell numbers **(D)** shows B-cell-numbers which are significantly depleted by all substances **(E)** the CD4/CD8-ratio which is affected by daratumumab and pomalidomide. All p-values were calculated in an exploratory manner using Wilcoxon’s signed rank test without adjustment for multiple testing. ** refers to <0.01 and *** refers to a p-value <0.001.

In order to assess the effects of individual substances on the development of CD4+-T-cell numbers after 3 months of relapse therapy (T2), an additional multivariable linear regression analysis with outcome log10(CD4+-T-cell numbers) was performed. Due to the low number of patients receiving elotuzumab (n=8) we did not specifically examine the effects of elotuzumab. Both carfilzomib and pomalidomide were associated with a significant decrease in log10 CD4+-T-cell numbers with an estimated effect of -0.18 (95CI [-0.32; -0.04]; Wald p=0.014) for carfilzomib and -0.26(95CI [-0.43; -0.09] Wald p=0.003) for pomalidomide respectively. Daratumumab was not associated with a statistically significant decrease (estimated effect of daratumumab -0.09; 95CI [-0.23; 0.04]; Wald p=0.17) (**Figure 3A** and **Tables 3**, **S5**).

**Table 3:** Results of the fitted linear regression model for log10 CD4+- T-cell counts at T2.

	Units	Coefficient	CI95	p-value
(Intercept)		0.84	[0.34;1.35]	0.001
Log_10_ CD4+-T-cells (T1)		0.70	[0.52;0.88]	<1e-04
Pomalidomide	no yes	Ref -0.26	[-0.43;-0.09]	0.003
Daratumumab	no yes	Ref -0.09	[-0.23;0.04]	0.17
Carfilzomib	no yes	Ref -0.18	[-0.32;-0.04]	0.01

Treatment with pomalidomide and carfilzomib are both associated with a decrease of CD4+-T-cells at T2 (p=0.003 and p=0.014 respectively) while daratumumab shows no statistically significant effect (p=0.17). VIF are shown in **Table S5**.

### Effects of therapy on other immune cell populations

In addition, we exploratively analyzed the effects of individual substances on further immune cell subpopulations. In carfilzomib and pomalidomide subgroups, no statistically significant difference in the development of CD8+-T-cells was detected. However, treatment with daratumumab led to a significant decrease of NK-cells (after 3 and 6 months, Wilcoxon signed p<0.001) in line with previous findings (32). Interestingly, all substances significantly (p<0.001 for all substances at T2, p<0.01 for daratumumab at T3 and p<0.001 for carfilzomib and pomalidomide at T3) decreased circulating B-cells over time, providing a potential further mechanism for impaired immune responses (**Figures 3B–E**, **S1**).

### Predictors of infectious complications

In order to identify factors contributing to susceptibility to infectious complications, we applied a continuation ratio model (38) In order to identify factors contributing to susceptibility to infectious complications in the first 3 months, we applied, adjusting for the predefined covariates age, corrected IgG (<4g/l vs ≥4g/l), previous lines of therapy and absolute CD4+-T-cell number at T1 (35). Lower CD4+-T-cell numbers were associated with a slight effect on the occurrence of infections (Est.=-0.0014) but failed to reach statistical significance (p=0.148) (**Table S6**). However, none of the other covariates reached statistical significance either.

## Discussion

Our study provides real world data on the development of immune cell subsets, in particular CD4+-T-cells in RRMM patients. We demonstrate that CD4+-T-cells are strongly depleted in a majority of patients already at the initiation of relapse therapy, linked to the number of previous lines of therapy. Notably, it was recently demonstrated that lines of therapy are also associated with increased risk for infection (39). In addition, we show that treatment with the novel agents pomalidomide and carfilzomib leads to further depression of CD4+-T-cells. Notably, all substances also demonstrated a significant negative effect on B-cell numbers, suggesting an additional mechanism of immunosuppression. We could not determine a direct link between CD4+-T-cell levels and infections in general in the setting of CD4+-T-cell-guided anti-infective prophylaxis strategies. However, our findings suggest an increased need for prophylaxis of opportunistic infections in RRMM patients.

CD4+-T-cells are a central link in the adaptive immune system with implications for opportunistic and viral infections, previously demonstrated for MM patients receiving traditional chemotherapy and bortezomib (6, 26, 28, 30). Identifying a substantial part of RRMM patients with CD4+-T-cell-numbers comparable to patients with AIDS stage III suggests an increased need for prophylaxis of opportunistic infections in this patient group (29). While broad prophylaxis for all RRMM patients can be considered, side effects such as decreased renal function caused by acyclovir and allergic reactions to cotrimoxazole should favor a risk stratified approach. Our study suggests that patients in later lines of therapy and patients receiving treatment with agents associated with further depletion of CD4+-T cells such as pomalidomide or carfilzomib might particularly benefit from prophylaxis against opportunistic infections.Also, determining CD4+-T-cell numbers before the initiation of relapse therapy may also help to better stratify patients requiring prophylaxis.

Notably, no patient had an absolute neutrophil count below 0.5/nl at initiation of relapse therapy. However, not only CD4+-T-cells were depleted by treatment with novel agents, but also other lymphocyte subtypes, such as B-cells. This might provide a rationale for the decreased efficacy of vaccinations in RRMM patients, as observed in the COVID-19 pandemic (17, 18, 40, 41). Lack of CD4+- and other T-cells might also explain the high risk for hospitalization and death due to COVID-19 (13, 30, 31).

Despite the severely decreased CD4+-T-cells, only one opportunistic infection (herpes zoster-reactivation) occurred during the observation period. This infection occurred in a patient who had a CD4+-T-cell count only slightly above 200/µl before the beginning of therapy, but who had subsequently fallen below 200/µl during therapy and had not received anti-zoster-prophylaxis in between. A potential explanation for the very low number of opportunistic infections may be that study patients with low CD4+-T-cell-counts (<200/µl) consequently received anti-zoster and anti-PcP prophylaxis according to guidelines (29), supporting the strategy of CD4+-T-cell guided prophylaxis. Given these results, we suggest anti-zoster and anti-PcP prophylaxis in all patients receiving carfilzomib or pomalidomide for at least 3 months. Given the high prevalence of depressed CD4+-T-cells in RRMM patients, prophylaxis against opportunistic infections should be generously considered in all RRMM patients.

Due to the real life setting and the limited number of patients, our study has some limitations. Unfortunately, we were not able to establish a link between CD4+-T-cell numbers and infections in general. While we did observe a small effect, this effect was not statistically significant. This may partially be due to our cohort size with potentially more patients and infectious events required to examine this effect in a multivariable analysis. Furthermore, since patients with seriously decreased CD4+-T-cells consequently received prophylaxis, this might mask stronger effects of very low CD4+-T-cell numbers. However, we appreciate that general infection risk may also be more complex than what can be measured with immune cell subtyping (42, 43).

Due to the high variability in treatments, we were also not able to assess the effects of individual substances on infection rates and whether there are synergistic or antagonistic effects on CD4+-T-cell numbers. We appreciate that larger studies are needed to address the question if particular novel agents or combinations increase the risk of infections, as has been implied for pneumonia and upper respiratory tract infections during treatment with daratumumab (44).

Future studies may also address the question over which time frame immunosuppression occurs. Our study implied that the most pronounced effect can be seen after 3 months, however recovery at 6 months implies, there might also be stronger effects before 3 months and potentially less effects at timepoints later than 6 months. It might also be of interest to specifically examine infections under treatment with pomalidomide and/or carfilzomib to see if a decrease in CD4+-T-cells also corresponds to increased infections in this particular subgroup.

Given the advent of novel T-cell redirecting treatments in MM, which directly depend on the state of the immune system, it would also be of interest to assess if the changes we observed also influence the efficacy of these treatments. This could have an impact on the choice of bridging therapies and might lead to better therapy sequences.

Taken together, our study provides novel insights into cellular immune defects caused by novel agents in RRMM patients in later lines of therapy. Our data provides a rationale for immune cell subtyping before the start of therapy to better guide prophylaxis of opportunistic infections in RRMM patients.

## Data availability statement

The raw data supporting the conclusions of this article will be made available by the authors, without undue reservation.

## Ethics statement

The studies involving human participants were reviewed and approved by Heidelberg University Hospital Ethics comittee no. S-096/2017. The patients/participants provided their written informed consent to participate in this study.

## Author contributions

CM-T, DK, LJ, EK-M, HG, KJ, NG and MSR provided study material and clinical data. KK and MH performed FACS analysis. KM and AB performed statistical analysis. EKM, KM, LJ and NG analyzed the data. LJ and NG designed the study and wrote the paper. All authors contributed to the article and approved the submitted version.
