# Supplementary material for: Impact of novel agent therapies on immune cell subsets and infectious complications in patients with relapsed/refractory multiple myeloma
Source: Front Oncol. 2023 Apr 21;13:1078725. doi: 10.3389/fonc.2023.1078725 (PMC10160457; doi:10.3389/fonc.2023.1078725)
Supplement: Supplementary file 1 [file DataSheet_1.docx]

Supplementary Material

**Supplement:**

| **Supplementary Table 1: Treatment Overview** | | | |
| --- | --- | --- | --- |
| **Treatment** | **T1** | **T2** | **T3** |
| Daratumumab/Dexamethasone | 3 | 2 | 2 |
| Daratumumab/Lenalidomide/Dexamethasone | 21 | 17 | 9 |
| Daratumumab/Lenalidomide/ Cyclophosphamide /Dexamethasone | 1 | 0 | 0 |
| Daratumumab/Pomalidomide/Dexamethasone | 3 | 3 | 2 |
| Daratumumab/Pomalidomide/ Cyclophosphamide/Dexamethasone | 1 | 1 | 1 |
| Daratumumab/Bortezomib/Dexamethasone | 26 | 23 | 15 |
| Daratumumab/Carfilzomib/Dexamethasone | 9 | 9 | 8 |
| Elotuzumab/Pomalidomide/Dexamethasone | 6 | 5 | 2 |
| Elotuzumab/Lenalidomide /Dexamethasone | 3 | 3 | 3 |
| Carfilzomib/Lenalidomide/Dexamethasone | 9 | 6 | 1 |
| Carfilzomib/Cyclophosphamide/Dexamethasone | 10 | 8 | 2 |
| Carfilzomib/Dexamethasone | 4 | 4 | 4 |
| Pomalidomide/Cyclophosphamide/Dexamethasone | 10 | 7 | 5 |
| Pomalidomide/Dexamethasone | 6 | 3 | 1 |
| Total | 112 | 91 | 55 |

**Supplementary Table 1:** Overview over individual treatment combinations at different timepoints

| **Supplementary Table 2: Reasons for loss of follow-up/discontinuation of therapy** | |
| --- | --- |
| **Reasons for discontinuation of therapy after T1** | **Number of patients**  **(total 21)** |
| Death due to progression | 8 (38.1%) |
| Progression | 4 (19.0%) |
| Others (change of therapy due to reasons other than progression) | 9 (42.9%) |
| **Reasons for discontinuation of therapy after T2** | **(total 36)** |
| Progression | 12 (33.3%) |
| Death due to progression | 2 (5.6%) |
| Others (change of therapy due to reasons other than progression) | 21 (58.3%) |
| Death due to infection (Influenza) | 1 (2.7%) |

**Supplementary Table 2:** Reasons for loss of follow-up and discontinuation of therapy during the study

| **Suppl. Table 3: Antimicrobial prophylaxis** | |
| --- | --- |
|  | **Number of patients** |
| **Ongoing therapy at T2** | 91 (81.3%) |
| Acyclovir prophylaxis (T1-T2) | 78 (84.5%) |
| PcP-prophylaxis (T1-T2) | 42 (45.7%) |
| Antibiotic prophylaxis (T1-T2) | 24 (26.1%) |
| Intravenous immunoglobulin substitution | 10 (11%) |
| **Ongoing therapy at T3** | 55 (49.1%) |
| Acyclovir prophylaxis (T2-T3) | 48 (87.3%) |
| PcP-prophylaxis (T2-T3) | 25 (45.5%) |
| Antibiotic prophylaxis (T2-T3) | 4 (7.3%) |
| Intravenous immunoglobulin substitution | 4 (7.3%) |

**Supplementary Table 3:** Overview over antimicrobial prophylaxis

| **Suppl. Table 4:** **VIF for main table 2** | |
| --- | --- |
|  | **Variance inflation factor** |
| Age | 1.01 |
| Active therapy within the last 6 months | 1.04 |
| Number of previous therapies | 1.05 |

**Supplementary Table 4:** **Variance inflation factors for main table 2.**

| **Suppl. Table 5: VIF for Table 3** | |
| --- | --- |
|  | **Variance inflation factor** |
| Log_10_ CD4+-T-cells (T1) | 1.11 |
| Pomalidomide | 1.95 |
| Daratumumab | 1.82 |
| Carfilzomib | 1.80 |

**Supplementary Table 5: Variance inflation factors for main table 3.**

**
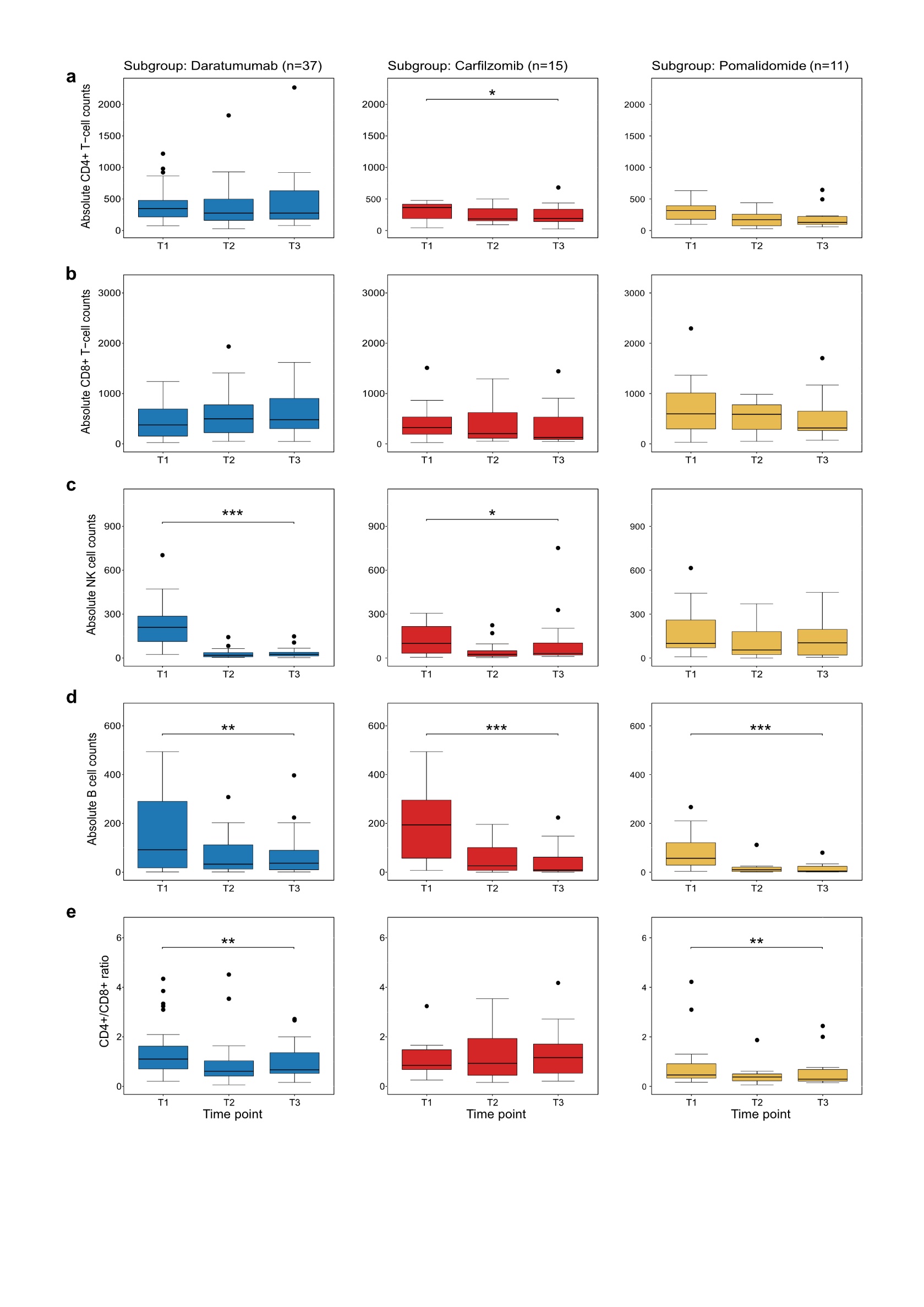
**

**Supplementary Figure 1:** **Descriptive analysis of individual novel agents on immune cell subsets** This figure shows the distribution of immune cell subsets in patients with data for all 3 timepoints (before start of therapy (T1), after 3 months (T2) and after 6 months (T3) for different treatment subgroups (a) shows the distribution of CD4+-T-cell numbers (b) shows CD8+-T-cell numbers with no substance showing a significant effect. (c) shows NK-cell numbers, again daratumumab significantly reduced NK-cell numbers (d) shows B-cell-numbers and which are significantly depleted by all substances (e) CD4/CD8-ratio is affected by daratumumab and pomalidomide. All p-values were calculated in an exploratory manner using the Friedman test without adjustment for multiple testing. * refers to a p-value <0.05, ** refers to <0.01 and *** refers to a p-value <0.001

| **Supplementary Table 6: Results of the continuation ratio model for absolute CD4+ T-cell counts at T2.** | | | | |
| --- | --- | --- | --- | --- |
|  | **Estimate** | **Std. Error** | **z value** | **Pr(>\|z\|)** |
| (Intercept) 1 | 3.8710 | 2.0343 | 1.9029 | 0.0571 |
| (Intercept) 1 | 5.0936 | 2.0885 | 2.4389 | 0.0147 |
| Absolute CD4+-T-cells (T1) | -0.0014 | 0.0010 | -1.4481 | 0.1476 |
| Age | -0.0212 | 0.0287 | -0.7378 | 0.4607 |
| Number of pre-therapies | -0.1459 | 0.1264 | -1.1547 | 0.2482 |
| Corrected IgG | 0.3718 | 0.5355 | 0.6943 | 0.4875 |

**Supplementary Table 6:** Results of the continuation ratio model for log10 CD4+-T-cell counts at T2. No covariate is significantly associated with infections. The p-value for CD4+-T-cells is 0.1476.
